# Seismic Behavior of Superelastic Shape Memory Alloy Spring in Base Isolation System of Multi-Story Steel Frame

**DOI:** 10.3390/ma12060997

**Published:** 2019-03-26

**Authors:** Yuping Liu, Hongyang Wang, Canxing Qiu, Xingnan Zhao

**Affiliations:** 1 School of Civil Engineering, Shandong University, Jinan 250061, China; lyplemon@sdu.edu.cn (Y.L.); wwanghongyang@163.com (H.W.); ZhaoXNan@yeah.net (X.Z.); 2 Key Laboratory of Urban Security and Disaster Engineering of Ministry of Education, Beijing University of Technology, Beijing 100124, China

**Keywords:** SMA spring, seismic isolation, cyclic loading tests, FE simulation, earthquake engineering

## Abstract

Owing to excellent re-centering capability and good damping behavior, superelastic shape memory alloys (SMAs) are emerging as a potential new material to enhance the seismic resilience of civil structures. This paper focuses on using base isolation with SMA device for isolated structures. SMA springs are deemed to be promising candidate as the damper in the base isolation system, due to the compact form, damping contribution, restoring capability and flexible stiffness. This paper reported the concept of an innovative spring which is made of superelastic SMA wire. Then cyclic loading tests were carried out to evaluate the interested cyclic properties. Parametric analyses based on finite element simulations were conducted to reveal the versatile performance of SMA springs. To further examine its seismic behavior in the base isolation system, the SMA spring was later installed at the isolation level of a multi-story steel frame, based on the finite element model built in the earthquake engineering simulation platform OpenSees. An ordinary elastic spring is included for comparison to highlight the features of SMA springs. Both isolated frames were subjected to real earthquakes. The comparisons indicated that using SMA spring is more effective in controlling maximum and residual deformation for the protected superstructures. Thus, this paper well demonstrated the feasibility and merits of using SMA springs in the isolated frames.

## 1. Introduction

Using seismic isolators has been found an effective way to protect low- to middle- rise structures under earthquakes in past investigations and practices [1]. According to the principle of structural dynamics, seismic isolators are required to have much smaller lateral stiffness than that of the protected superstructure. Although adding stiffness or damping at the isolation level is not suggested from the viewpoint of protecting the superstructure [2], small stiffness and damping may result in excessive deformation at the isolation level toward the earthquake attack direction. In the practical applications, the allowable deformation space for isolation system is usually limited by the adjacent structures or foundations. In fact, as reported in the Northridge earthquake [3], the limited isolation gap generated suddenly increased shear force and interstory drift in the superstructure as a result of the pounding impact. Therefore, it is advisable to achieve a balance between controlling deformation for the isolators and alleviating the counter effect of increasing seismic demand in the superstructure.

**Figure 1:**
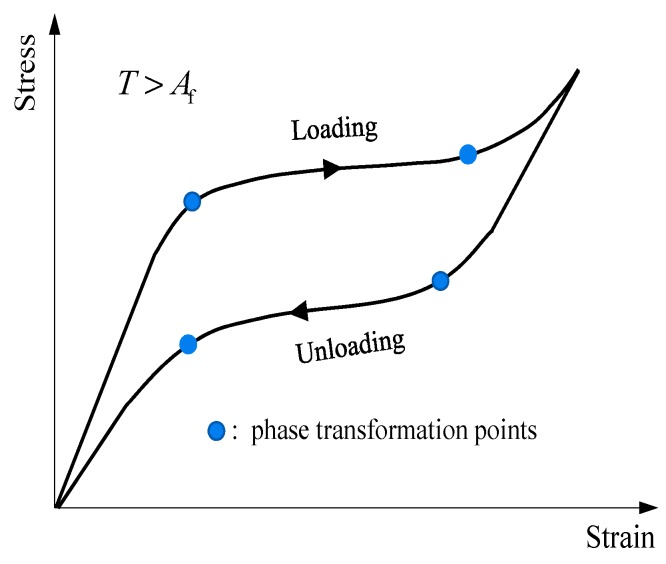
Cyclic behavior of superelastic shape memory alloys (SMAs).

In this context, various damping devices have been proposed to assist the isolators to deform in allowable space by absorbing the input seismic energy [4,5]. Representatives primarily include viscous damper [6], friction damper [7] and lead damper [8]. But these dampers are found tend to shift superstructures away from the initial position. Therefore, besides with controlling peak deformation demand, the residual deformation of the isolation level should be minimized as well, with the aim to restore the structure to its at-rest position. To fulfill this requirement, people made encouraging attempts in developing advanced isolators, such as the friction-pendulum base isolator [9] and conical spring isolator [10]. Recently, to address the issue, the research community resorted to incorporating shape memory alloys (SMAs) into the isolation systems. SMA is a class of metal materials, which are able to exhibit excellent superelasticity when the environmental temperature is above the phase transformation threshold [11]. This feature is attributed to the solid-to-solid transformation between two crystallographic phases, namely, austenite and martensite [12,13]. Phase transformation can be activated by varying ambient temperature or changing stress state. The former is referred to shape memory effect and the latter is known as superelastic effect. In seismic applications, the superelastic effect is of particular interest. As can be seen in Figure 1, the loading and unloading behaviors of superelastic SMAs are triggered by recoverable phase transformation of crystalline, forming a flag-shape hysteresis.

The studies of using SMA devices in the base isolation system of buildings are getting increasing attentions. For example, Ponzo et al. [14] compared three different isolation systems through shaking table tests and found that the SMA damper is better than the counterparts. Gur et al. [15] focused on near-fault seismic performance and indicated the enhancement of the isolation efficiency provided by SMA was outstanding in suppressing the acceleration and displacement demands. Ozbulut and Silwal [16] used multi-objective genetic algorithm to optimize the design parameters for SMA damping isolator. Qiu and Tian [17] considered the strain hardening behavior of the SMA damper in particular and highlighted the advantage of this behavior in controlling deformation demand. A proof-of-concept shake table test for shear frames isolated with SMA springs was carried out by Huang et al. [18]. Attanasi et al. [19] investigated the behavior of a base isolated building using conventional lead rubber bearing or SMA devices. Although the efficacy of SMA device was shown, the superstructure was not explicitly modeled in the seismic analysis, which thus ignored the seismic behavior of the superstructures. With the noticeable achievement made so far, it is still worth noting that past studies are often based on the assumption that the superstructure is always in its elastic stage. However, this assumption is actually over idealized, because the superstructure is likely to endure nonlinear deformation under moderate or large earthquakes.

In this study, SMA spring damper is proposed to be implemented in the isolation system for frame structures. Compared with prior studies [17,18,19], the building nonlinearity was well modeled and discussed in the current analysis. According to the ductile design philosophy, structures are usually permitted to deform into inelastic state by moderate and severe earthquakes. Therefore, with considering the nonlinear behavior of the superstructure, the seismic performance of the structures isolated with SMA springs can be more realistically revealed. The advantages of SMA spring dampers include the concise form, stand-alone property, the extraordinary deformation capability through transforming axial deformation into shear deformation, the versatile performance by tuning the design parameters to achieve desired properties. Cyclic loading tests were carried out to assess the seismic properties of the SMA spring. Then, finite element analyses were conducted to quantify the effect of varying geometrical dimensions on the mechanical properties of SMA springs. Finally, a prototype nonlinear multi-story frame is selected in the seismic analyses, with the aim of further revealing the feasibility of using SMA springs in the isolation system of frame buildings.

## 2. SMA Spring Damper

### 2.1. Manufacturing Process

**Figure 2:**
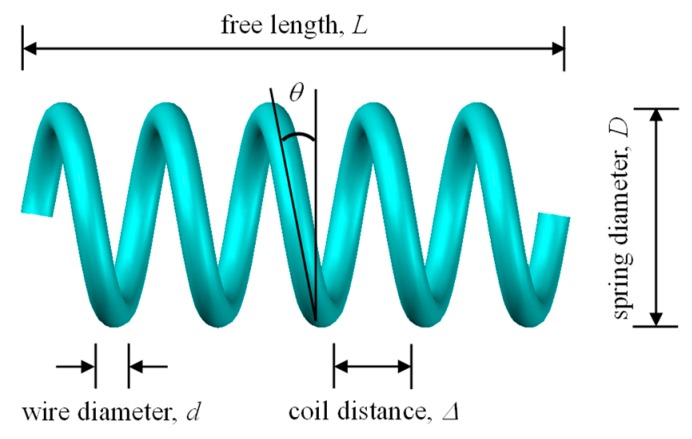
Geometric dimensions of a typical spring.

Due to several merits, including large re-centering strain, good energy dissipation, high fatigue life and outstanding corrosion resistance, NiTi SMA is widely explored in current seismic application research [20]. Thus, NiTi SMA wire is selected to fabricate the spring damper. Like the ordinary elastic springs, the cyclic properties of SMA springs also depend on various parameters, which allows the designers high flexibility to select suitable spring configurations to meet the practical requirements. This section presents seismic application-oriented characterization of SMA springs through cyclic loading tests. Figure 2 shows the notation of typical geometric dimensions of a spring. As can be seen, a total of four geometric parameters are usually included in the design of a helical spring: wire diameter *d*, spring diameter *D*, pitch angle *θ* and the number of active coils *N* that is equal to the ratio of free length to coil distance, that is, *L*/Δ. Besides, a spring index is defined as *C* = *D*/*d*. In this study, the NiTi SMA wires were purchased from Xi’an Saite Metal Material Development Co. Ltd. in Xi’an, China. According to the supplier, the chemical composition of this slot of NiTi SMA is 56.01% Ni and 43.00% Ti in weight percentage. The austenite finish temperature is Af = 0 °C. The parameters *D*, *d*, *C*, *N*, Δ and *L* are 16, 2, 8, 6, 8 and 62 mm, respectively.

**Figure 3:**
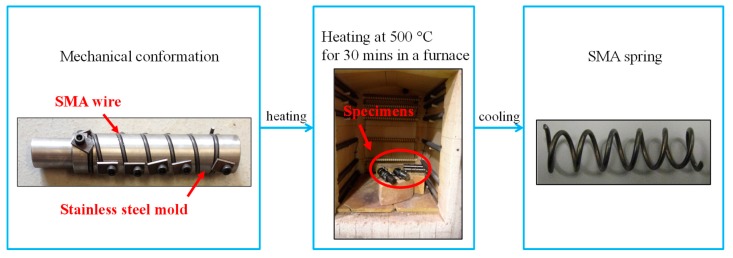
Procedures of fabricating SMA springs.

Because of the complex thermo-mechanical behavior of SMA material, it is usually very challengeable to manufacture satisfactory superelastic SMA springs. For example, the manufactured SMA springs by a prior study did not show “yielding” plateau [18]. To obtain desirable cyclic behavior of SMA spring, the manufacturing procedure is searched through several trial-and-error attempts. Thus, we decided to report the manufacturing procedure. The SMA spring was fabricated in the laboratory. Since SMAs are temperature-dependent material, proper heating treatment is a critical step in the manufacturing process. In fact, it has been recognized that heating treatment process affects the mechanical properties of SMA springs [21]. Morgan and Broadley [22] tried various heating temperatures and they recommended the proper temperature is between 450 °C and 550 °C. Jee et al. [23] heated the specimens to 550 °C for 30 min. Recently, Savi et al. [24] confirmed heating treatment at 500 °C for 30 min is the best way to make an ideal SMA spring after a serious of trials. To find an optimal heating method, this study tried three temperatures: 300 °C, 400 °C and 500 °C and three heating treatment durations: 5 min, 10 min and 30 min. After the trials, the heating treatment at 500 °C for 30 min is found one potential option for producing desirable superelastic behavior. Water or air quench of the spring specimens were conducted after heating. In summary, the SMA springs are fabricated in the following three steps: (1) mechanical conformation of the springs; (2) 30 min heating treatment at 500 °C in a furnace; (3) air or water quench over a sufficient time. The procedures are shown in Figure 3.

### 2.2. Test Results

**Figure 4:**
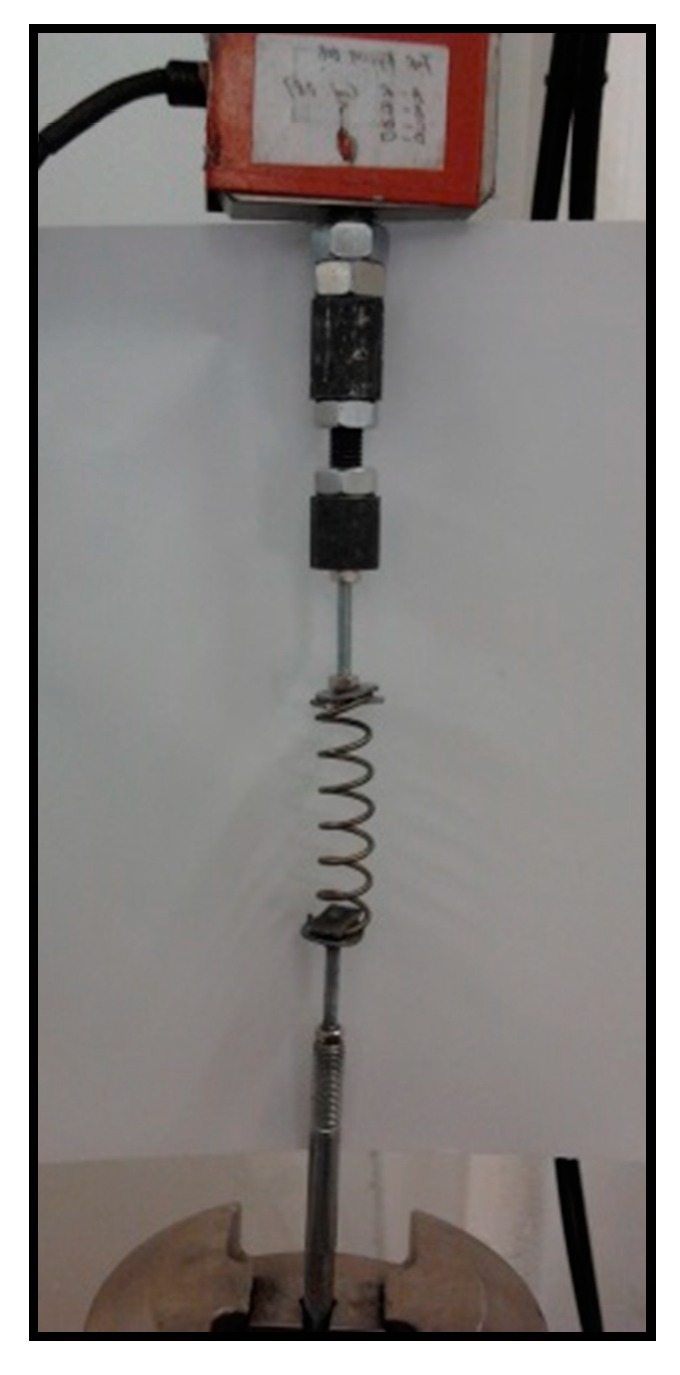
Experimental setup and tested specimen.

**Figure 5:**
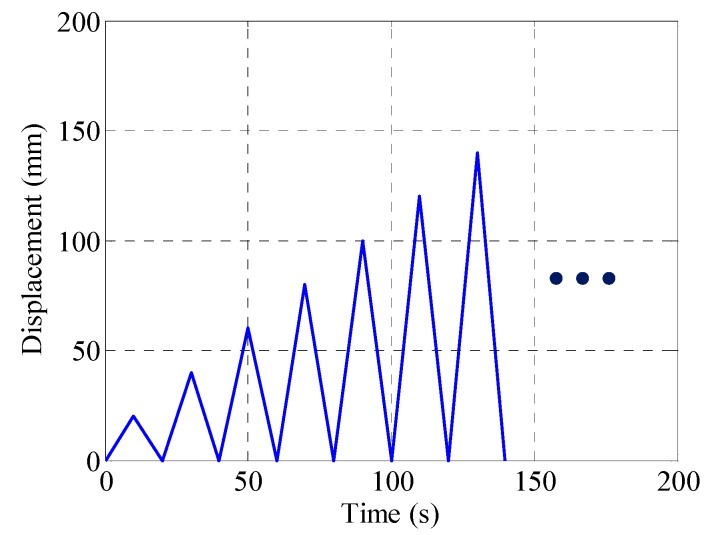
Representative cyclic loading protocol (loading frequency = 0.025 Hz).

The cyclic loading tests of spring specimens were conducted on an MTS universal testing machine (MTS Systems Corporation, Shanghai, China) at room temperature. Both specimen ends were clamped by two steel shims bolted to rigid fixtures, as shown in Figure 4. The cyclic tensile tests were conducted using the displacement control method. Figure 5 shows a representative quasi-static testing protocol in which the displacement amplitude keeps increasing with an increment of 20 mm until either the specimen exhibits noticeable residual deformation, or the displacement amplitude exceeds the loading limit of the MTS machine. In fact, the loading procedure was stopped at 140 mm, which is large enough to reflect the typical nonlinear behavior of the manufactured SMA spring.

**Figure 6:**
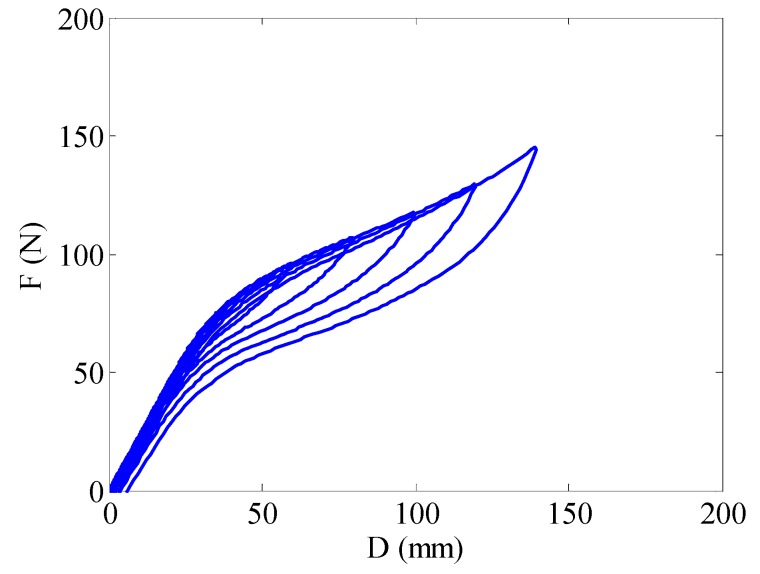
Cyclic behavior of the SMA spring.

Figure 6 shows the force-displacement relationship of the fabricated SMA spring obtained in cyclic tensile tests. As aforementioned, the maximum displacement amplitude was 140 mm. The “yielding” strength and spring stiffness are characterized as F_y_ = 63.3 N and K_i_ = 1.97 N/mm, respectively. As expected, the strength and stiffness of springs is much lower than that of wires. The “post-yield” stiffness ratio, α, and the energy dissipation parameter, β, defined according to Christopoulos et al. [25], are approximately estimated to be 0.29 and 0.5, respectively. The cyclic behavior of SMA spring shows typical flag-shape hysteresis. Unlike conventional elastic springs, the SMA springs show highly nonlinear behavior. The deformation of SMA springs is essentially a summation of the elastic deformation and the martensite transformation induced deformation [26]. Stable and repeatable hysteresis loops without obvious training effect are observed. Training effect is usually exhibited by the Ni-Ti wires in initial several loading cycles [27], while the elimination of this effect in springs is possibly attributed to the heating treatment [28,29]. The “yielding” behavior is defined to occur at the point where the tangent stiffness starts to decrease. According to this definition, the “yielding” displacement occurs almost constantly at 32.1 mm.

**Figure 7:**
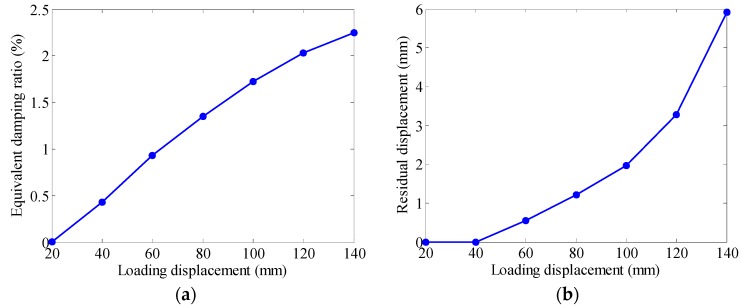
Loading amplitude effect on (**a**) equivalent damping ratio and (**b**) residual displacement.

Figure 7 plots the equivalent damping ratio and residual displacement as a function of loading amplitude. It is noted that the dissipated energy only considers the area in the first quadrant for the current spring. With proper configurations [30,31], the dampers using SMA springs are able to sustain both tension and compression actions. As shown in Figure 7a, the damping of SMA spring is relatively small, laying within the range of 1% to 3%. It increases with the loading amplitude, due to the gradual development of martensite fraction in SMA material. The martensite phase transformation also causes the accumulation of residual displacement with the loading amplitude. Figure 7b shows that, at the 140 mm loading loop, only 6 mm residual displacement is detected, which essentially implies the excellent re-centering capacity of SMA springs.

## 3. Finite Element Simulation in ABAQUS

This section conducted numerical investigation on the cyclic behavior of SMA springs in the general nonlinear finite element (FE) analysis program ABAQUS (Dassault Systèmes SIMULIA Corp., Providence, RI, USA). The purpose is to analyze the development of stress and strain in SMA spring under seismic loading scheme. A high-fidelity three-dimensional FE model will be built to further explore the cyclic behavior of the test specimen. The FE model was firstly built and calibrated by the test results. Then parametric analyses were carried out on wire diameter and spring diameter, with the purpose of demonstrating the versatile hysteretic properties of SMA springs through varying the geometrical dimensions. The corresponding outcome helps to shed light on the seismic design of SMA springs. This section is to demonstrate the versatile hysteretic properties of SMA springs through varying the geometrical dimensions. In practice, to scale up the strength of isolators, there are many ways including using dozens of springs in parallel, utilizing SMA bars with large diameter and change the geometrical dimension of the SMA springs.

### 3.1. Model Calibration

**Figure 8:**
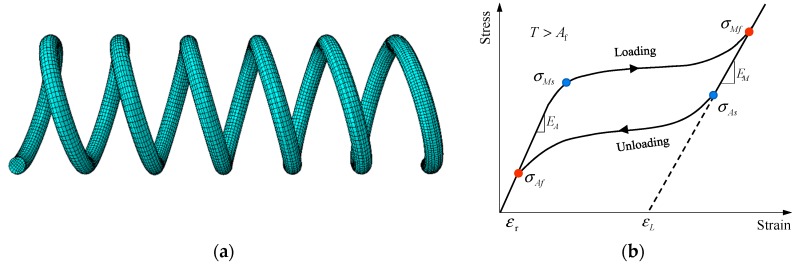
(**a**) Finite element model of SMA spring and (**b**) the constitutive model.

As can be seen in Figure 8, 3D FE model was built for the SMA spring. To improve the accuracy of the numerical simulation, the SMA wire was used the eight-node solid element and hourglass control (C3D8R). Considering that the meshing of component has a great influence on the efficiency and accuracy of numerical calculations, the final mesh of the spring was finely and regularly divided as shown in Figure 8. The meshing size is 0.35 mm, making at least 5 layers of elements throughout the thickness of the spring, which is adequate for capturing the complex stress distribution within the SMA spring. The loading scheme and boundary conditions of the FE model were as the same as that adopted in the test. To simulate the loading process, the spring is fixed at one end and the other end is loaded with an axially cyclic displacement load.

### 3.2. Material Properties

To simulate the behavior of SMAs, the superelastic material model implemented in ABAQUS was used. The stress-strain relationship of the NiTi SMA at room temperature was defined according to the parameters listed in the Table 1. The used material parameters stem from experimental data. E_A_ is the austenite elastic modulus; E_M_ is the martensitic elastic modulus; σ_Ms_ and σ_Mf_ are forward transformation stress featuring the start and complete of austenite to martensite phase transformation; σ_As_ and σ_Af_ are reverse transformation stress featuring the start and complete of martensite to austenite phase transformation; ε_t_ is the maximum transformation strain.

### 3.3. Simulation Results

**Figure 9:**
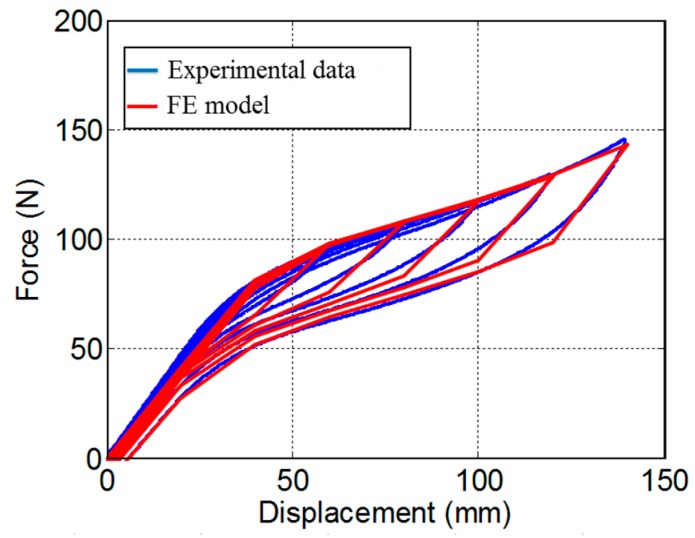
Comparison between the FE results and experimental data.

**Figure 10:**
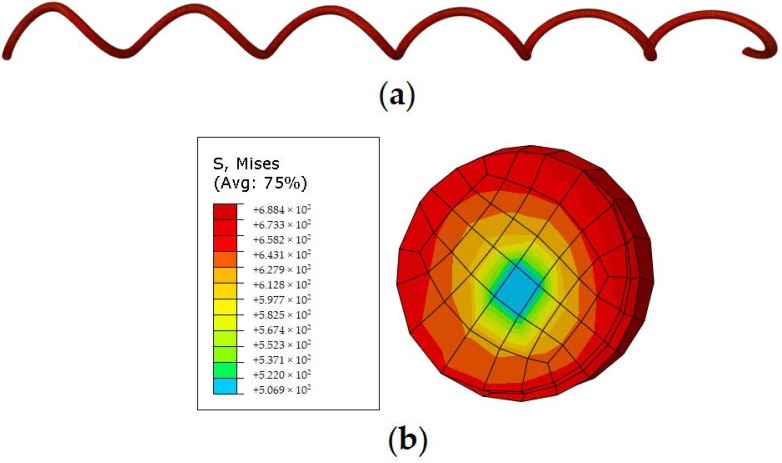
Mises stress distribution of deformed SMA spring: (**a**) global view; (**b**) cross section.

Figure 9 compares the force-displacement relationships of the SMA spring between the experimental data and FE simulated results. It can be seen that the FE model successfully reproduced the cyclic behaviors of the spring in every single loading cycle. The “yield” force and elastic stiffness are measured as approximately F_y_ = 65.6 N and K = 2.01 N/mm, the “post-yield” stiffness coefficient α and energy dissipation coefficient β are quantified to be 0.28 and 0.5, respectively, both of which agree with the test results very well, indicating that the established FE model captures the critical hysteretic parameters. Compared with test results, the FE model generate a relatively sharp phase-transformation process, which is primarily due to the simplification of the built-in SMA material model. Figure 10 shows the Mises stress state of the SMA spring when it was tensioned to a displacement of 140 mm. As can be seen from Figure 10a, the spring is uniformly stretched, and the tensile stress is evenly distributed throughout the body. To observe the internal stress of the specimen, Figure 10b presents the cross-sectional stress distribution. It is found that the maximum stress occurred at the edge of the cross section, reaching to approximately 688 MPa, which is larger than the forward phase transformation stress, indicating the local area has been partially deformed into martensite phase; while the stress level at the core of the cross section is approximately 500 MPa, which is smaller than the forward phase transformation stress, implying the core is still in austenite phase. The stress distribution is mainly caused by torsion and bending actions at the section.

### 3.4. Parametric Analysis

Based on the calibrated FE model established in above section, this section further conducts parametric analysis to examine the effect of varying the geometrical dimension on the cyclic behavior of SMA springs. The considered geometrical dimensions include the wire diameter and the spring diameter, as listed in Table 2. The parametric analysis also aims to demonstrate the versatile feature of SMA springs. To quantify the effect of varying the selected parameters, the interested hysteresis indices include the peak strength, tangent stiffness, equivalent damping ratio and residual displacement corresponding to each loading cycle.

**Figure 11:**
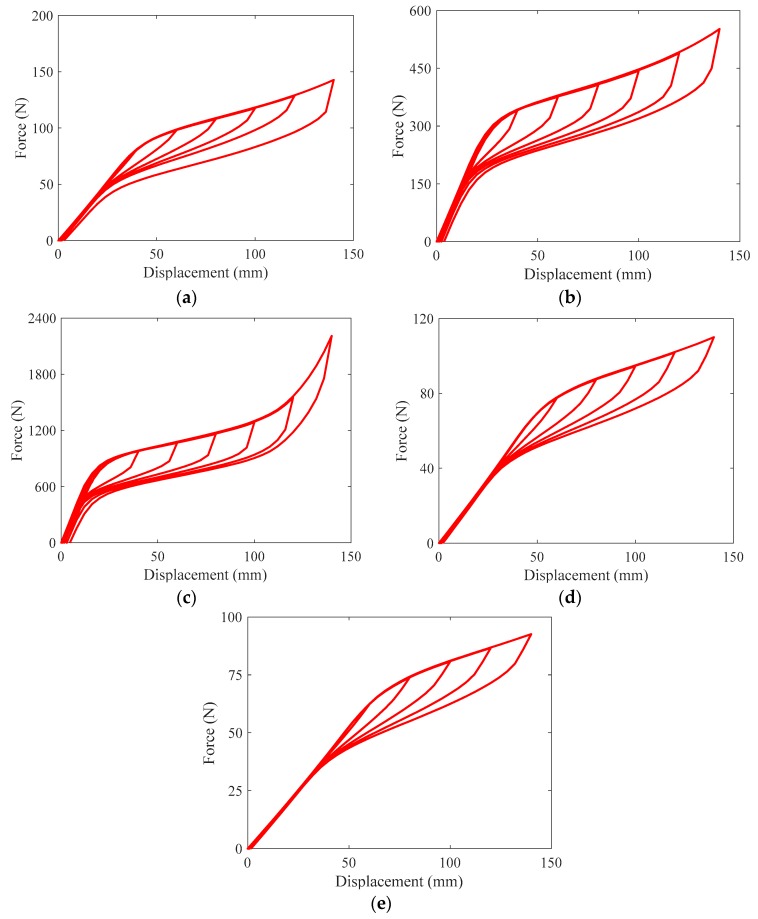
Effect of varying geometrical dimensions on cyclic behaviors of SMA spring: (**a**) S1; (**b**) S2; (**c**) S3; (**d**) S4; (**e**) S5.

Figure 11 assembles the hysteresis loops of SMA springs with different geometrical dimensions. The hysteresis of S1 is repeated here for reference purpose. To ensure a fair and direct comparison between these springs, the applied loading history is identical to that used in the experimental tests. Overall, it can be seen that the SMA springs always attain typical flag-shape hysteresis within the applied loading amplitude, although the wire diameter and spring diameter are severely changed. Specifically, when the wire diameters of S2 and S3 were respectively increased to 3 and 4 mm and the “yield” strength of the springs remarkably reach to 337 and 822 N, which are approximately 5 times and 12 times that of the peak force of S1. Compared with S1, the spring diameter of S4 and S5 was respectively increased to 18 and 20 mm and it is seen that the peak force of the spring was reduced to 61 and 59 N, which are approximately 93% and 89% of the peak force of S1. Therefore, it shows that the strength demand of the SMA spring can be conveniently tuned by varying the wire diameter or the spring diameter.

The initial stiffness of the SMA spring is estimated as well, since this index directly determines the fundamental period of the isolated structures. According to Liang and Rogers [26], the elastic stiffness of a spring can be expressed by the following equation:

*k* = (*Gd*)/(8*C*^3^*N*) (1)

where *G* is the shear modulus of SMA material, *d* is the wire diameter, *D* is the spring diameter, N is the number of active coils and *C* is the spring index, defined as *C* = *D*/*d*. It is seen that the initial stiffness of the SMA spring is proportional to *d*^4^ and inversely proportional to *D*^3^. The calculated stiffness of S1–S5 is listed in Table 3. Comparisons are made between the theoretical and numerical results, showing a maximum error of 6.9%. This validates the FE model again. Compared with S1, the stiffness of S2–S5 is 5.3, 16.8, 0.7 and 0.49 times that of S1, respectively, indicating the initial stiffness performs with a similar trend as the elastic strength capacity.

**Figure 12:**
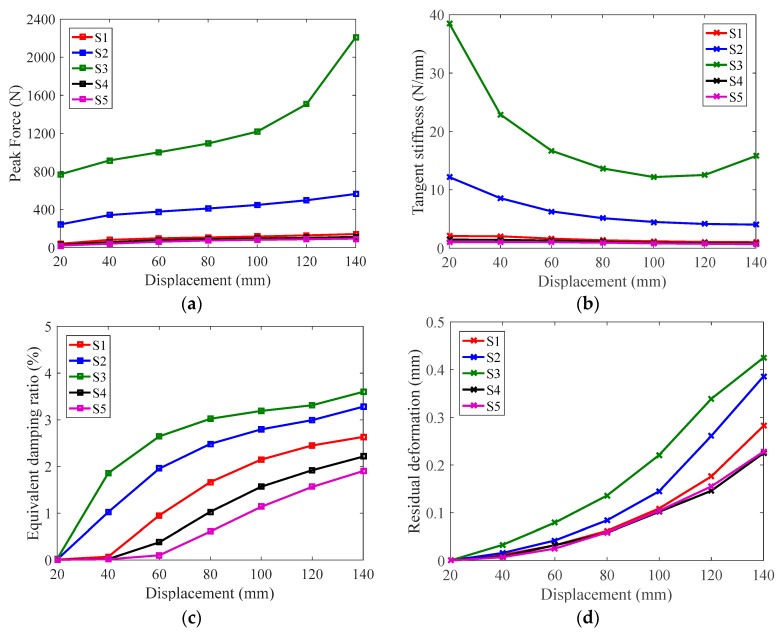
Critical hysteresis indices of S1–S5: (**a**) peak strength; (**b**) tangent stiffness; (**c**) equivalent damping ratio; (**d**) residual displacement.

The peak strength, tangent stiffness, equivalent damping ratio and residual deformation as a function of the applied displacements are shown in Figure 12. Figure 12a is about the peak force generated in each cycle. S3 has noticeably higher strength capacity than the others, primarily because of the large wire diameter directly increases lateral stiffness, which can be estimated by Equation (1). Figure 12b plots the tangent stiffness for each cycle. Again, S3 with a larger wire diameter exhibits higher stiffness. Figure 12c shows the variation of the equivalent damping ratio, it can be seen that the equivalent damping ratio increases with the displacement increment. The largest equivalent damping ratio is attained by S3 with a value of 3.6%; and the smallest value is found in S5. Figure 12d shows the residual deformation. Although the residual deformation tends to increase with the loading amplitude, the overall residual deformation is essentially very small, compared with the applied displacement. Among them, due to the strain hardening caused by the martensite phase, the largest residual deformation is generated by S3 with only 0.425 mm, which is low to 0.3% of the loading amplitude. Through the parameter analysis, it can be seen that adjusting wire diameter and spring diameter of SMA spring can effectively change the critical seismic performance indices of the damper and thus it allows the designers to properly tune the parameters to satisfy the requirements of seismic design.

## 4. Seismic Analyses

### 4.1. Building Information

**Figure 13:**
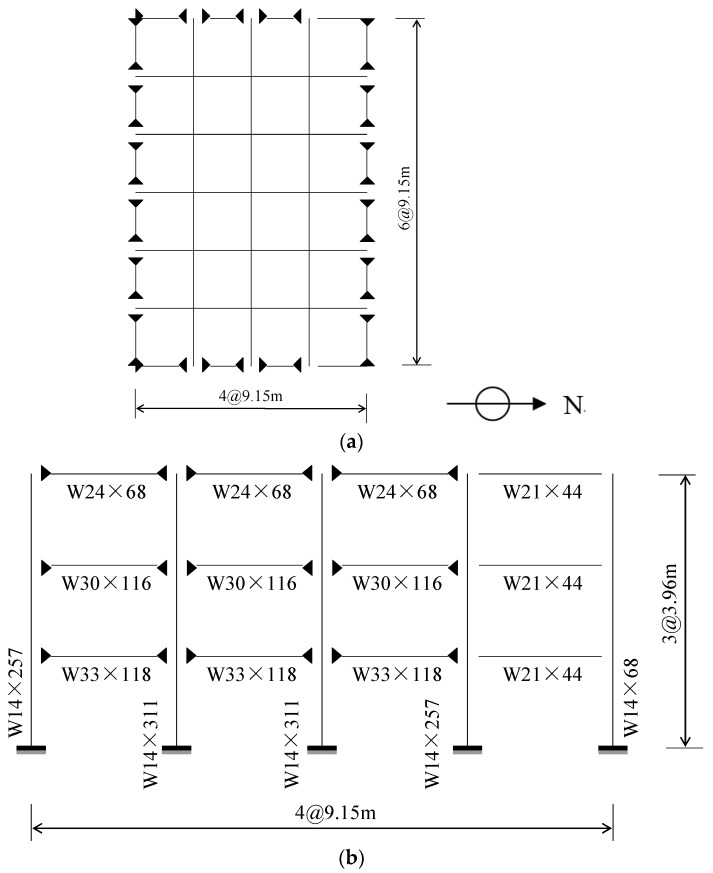
Plan layout and elevation view of the 3-story frame. (**a**) Plan layout; (**b**) elevation view.

In order to evaluate the seismic behavior of using SMA springs in the isolation system of multi-story steel frame under real earthquakes, a prototype 3-story frame is adopted for demonstration purpose. This frame was originally designed for the SAC (Note: SAC is a joint venture of three nonprofit organizations: The Structural Engineers Association of California (SEAOC), the Applied Technology Council (ATC) and California Universities for Research in Earthquake Engineering (CUREE)) project by Ohtori et al. [32]. The shape sections of the beams and columns are listed in Table 4. As shown in Figure 13a, the plan layout of the structure includes four bays in the north-south (N-S) direction and six bays in the east-west (E-W) direction. The bay width is 9.15 m in either direction. The columns and beams are made of steel with yielding stress of 345 and 248 MPa, respectively. Each floor is 3.96 m in height. The seismic mass of the first and second levels is 9.57 × 10^5^ kg and the third level 1.04 × 10^6^ kg. The shape sections of the beams and columns are shown in Figure 13b. To underscore the nonlinear characteristics of the SMA spring, the ordinary spring with elastic behavior is analyzed as well. It is worth noting that, to save the simulation effort in the seismic analysis, truss components are used to represent the real springs.

### 4.2. Design of the Springs

Prior to the seismic analyses, both the SMA and ordinary springs should be designed. Due to the missing of design guidelines for SMA-based isolators, the current design is based on the premise that the limitations of isolation gap are the same for both isolated structures and the designed springs will be deformed to the gap limitations under earthquakes. Based on the dynamic principle of isolated structures, the lateral stiffness of the isolation system should be much smaller than that of the superstructure. Thus, the first-mode pushover analysis was firstly conducted to obtain the initial stiffness and yielding strength of the superstructure and then the corresponding mechanical properties of the SMA spring are artificially set to be 10% that of the fix-base frame. The material and geometrical information for the SMA and ordinary springs are listed in Table 5. It is noted that the material properties of SMA is taken from prior tests, while those for ordinary springs are artificially assumed and dependent on ground motions. Since the springs were represented by truss elements, the corresponding cross-sectional area can be readily calculated. For the ordinary spring, the length is defined equal to that of the SMA spring and the elastic modulus is assumed to be that of steel. The cross-sectional area of ordinary springs is searched through trial-and-error iterations until the SMA and ordinary springs endured identical peak deformation demands. As a result, such design makes sure the isolators of both structures are avoided from pounding action during earthquakes. Since the isolation level will sustain the same deformation demands, such design idea helps to better focus on how will the adopted springs affect the seismic performance of the protected superstructures.

### 4.3. Numerical Model

**Figure 14:**
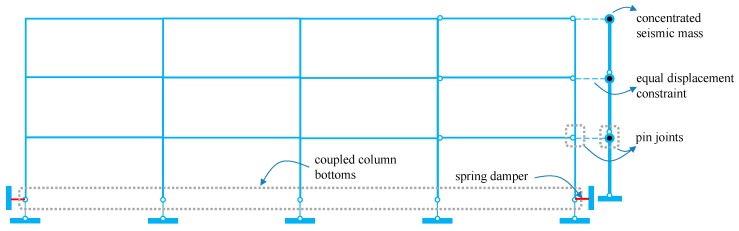
Numerical model of the 3-story frame built in OpenSees.

The numerical model of the three-story isolated frame was established in the earthquake engineering simulation platform OpenSees [33], as shown in Figure 14. A total of two numerical models were built, including the frames isolated with SMA springs or ordinary springs. Due to symmetry, only one half of the building was built, so the seismic tributary mass is 1/2 of the total floor mass. The model consists of a four-bay frame and a leaning column that are coupled at each floor level to undergo equal displacement demands. The building columns are continuous over height and are vertically supported at their bottoms, whereas the horizontal deformation is controlled by the springs. All the column bottoms are coupled together to sustain same deformation demand, which well represents the rigid isolation level. A leaning column carrying the effective seismic mass at each floor was built beside the frame and the adjacent two stories are connected by pinning, which is responsible for generating the P-Δ effect while avoiding lateral stiffness and strength to the entire structure. Springs are placed at the bottom of the outermost two columns to dissipate seismic energy and control seismic displacements within allowable gap. All beams and columns are modeled using force-based beam-column elements and springs are modeled with truss elements. The main frame, ordinary spring and SMA spring are designated with *Steel02*, *Elastic Uniaxial* and *Self Centering* materials, respectively.

### 4.4. Earthquake Ground Motion Records

**Figure 15:**
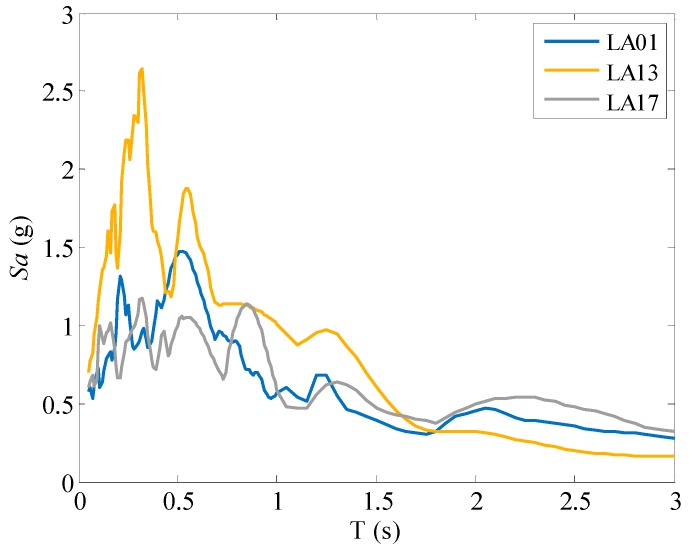
Spectral acceleration demand of 5% damping single-degree-of-freedom system under ground motion records LA01, LA13 and LA17.

To demonstrate the seismic performance of the isolated frames under earthquakes, in the seismic analyses, three earthquake ground motion records were selected from the suite of ground motion records developed by Somerville et al. [34], including LA01, LA13 and LA17. These ground motions represent the seismic hazard level corresponding to design-basis earthquake and their information are listed in Table 6. Figure 15 plots the spectral acceleration demands of 5% damping single-degree-of-freedom systems. The physical meaning of this figure is the maximum absolute accelerations incurred to the single-degree-of-freedom systems with a period of *T* and a damping ratio of 5%. As can be seen, these records show noticeable response variety.

### 4.5. Behavior of the Springs

**Figure 16:**
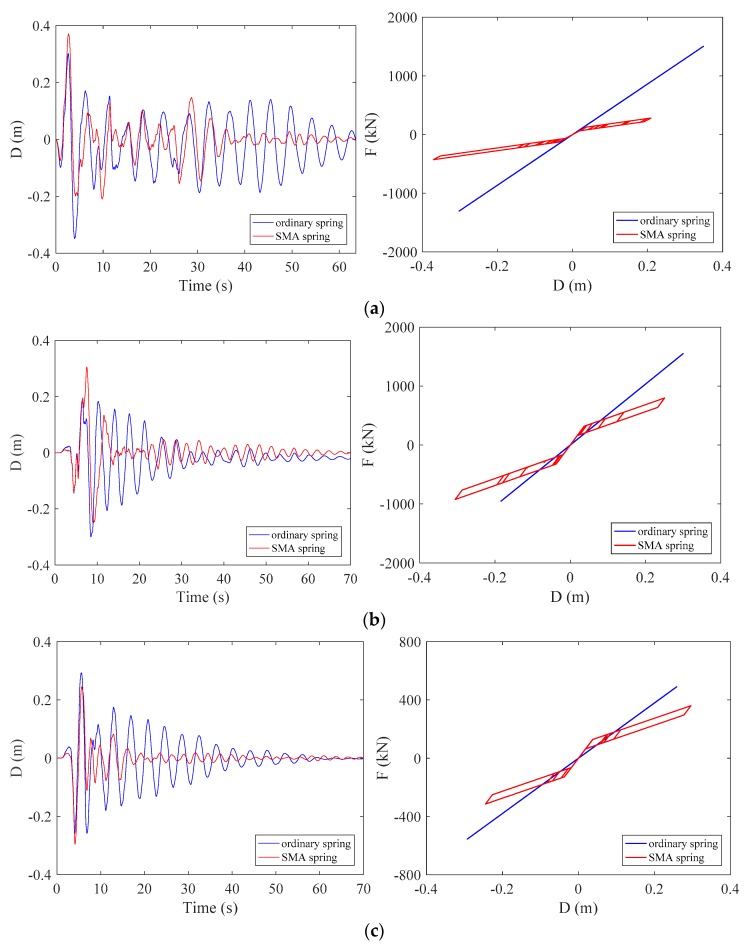
Displacement time histories and cyclic behaviors of the springs under selected earthquake ground motion records: (**a**) LA01 (**b**) LA13 (**c**) LA17.

Figure 16 shows the displacement time histories and cyclic behaviors of the two springs under three ground motions. The peaks of the displacement time history curves show these springs experienced same deformation demands. But using SMA springs leads to advantage over using ordinary springs. Thanks to the damping mechanism, the vibrations of SMA springs damped out more quickly than ordinary springs. The cyclic behaviors show noticeable difference between the adopted springs. As expected, the ordinary springs exhibit perfectly linear elastic behavior, while the SMA springs endured significant nonlinear deformation and exhibited typical flag-shape hysteresis. As can be seen, the peak deformations of the two springs are exactly the same but the ordinary springs generate noticeably larger force than SMA springs. The low strength demand of SMA spring is primarily attributed to the energy dissipation capacity, because increasing strength or damping is beneficial to control deformation demands. Thus, the strength is essentially compensated by adding damping capability. As a result, the ordinary springs would generate larger strength demand to the building columns and foundations than SMA springs.

### 4.6. Roof Responses

**Figure 17:**
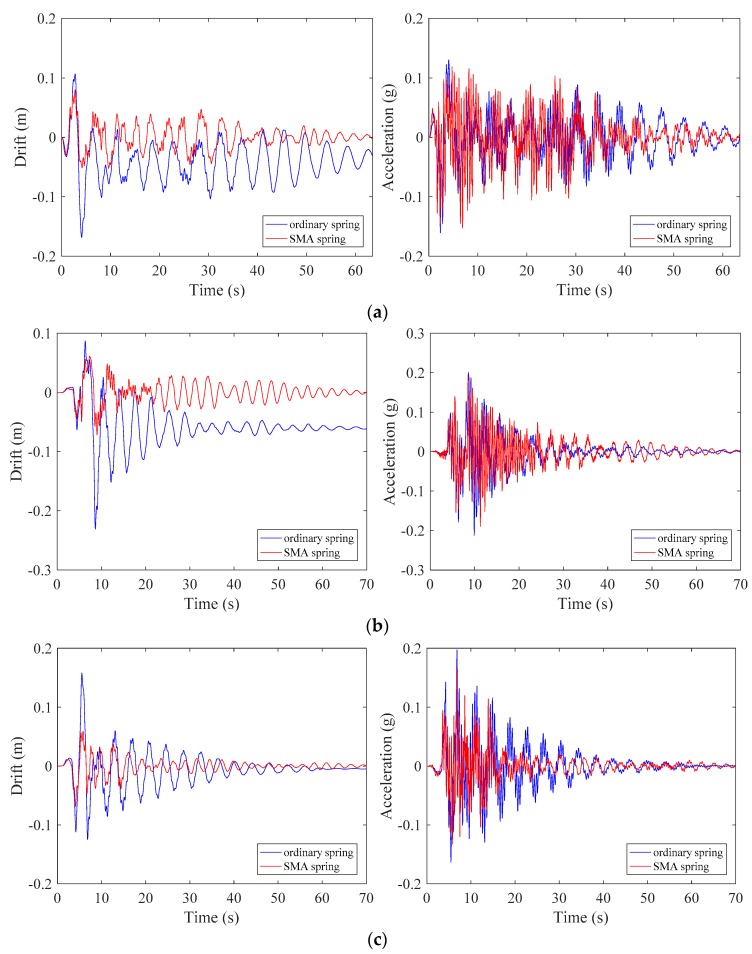
Roof responses of the isolated frames: (**a**) LA01; (**b**) LA13; (**c**) LA17.

To observe the global seismic performance of the isolated structures, Figure 17 plots the time histories of the roof drift and roof acceleration for the isolated frames subjected to the selected ground motion records. The term roof refers to the upper part of the third floor of the structure which is shown in Figure 13b and Figure 14. It is observed that SMA spring controlled roof displacement much better than ordinary spring in all three cases. Besides the mitigation of peak response, the building vibration is also damped out in a very short duration when SMA spring is installed. Further, at the end of the roof drift responses, the frame isolated with SMA springs left minimal residual deformation, whereas the counterpart suffered from noticeable residual displacement. In terms of roof accelerations, it seems insensitive to the adopted spring types and achieved similar performance over time.

### 4.7. Peak Interstory Drift Ratio

**Figure 18:**
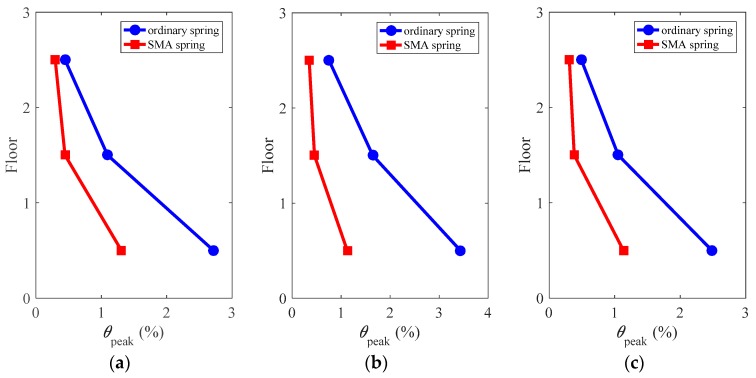
Peak interstory drift ratio of the superstructure: (**a**) LA01; (**b**) LA13; (**c**) LA17.

Figure 18 shows the peak interstory drift ratios of the superstructure along building height, defined as:

(2) $$\theta_{peak}=\max\left(abs\left(\theta_{i}\left(t\right)\right)\right),\;i=1,2,...,6$$

where *θ_i_* is the interstory drift ratio in the *i*th story during the total time duration of *t*.

It can be seen from Figure 18 that, under all considered ground motions, the peak interstory drift ratios tend to concentrate at the first story but the deformation concentration degree is well reduced by using SMA springs. Compared with ordinary springs, SMA springs offer better deformation control effect throughout building height. Specifically, under the ground motions of LA01, LA13 and LA17, the peak interstory drift ratios of the frames isolated with ordinary springs are up to approximately 2.7%, 3.4% and 2.5%, respectively, while the frames isolated with SMA springs are approximately 1.3%, 1.1% and 1.1%, respectively. The corresponding reductions are estimated to be over 50% in all cases. This clearly demonstrates the effectiveness of using SMA springs in the isolation system of the multi-story frames compared to ordinary springs.

### 4.8. Peak Floor Acceleration

**Figure 19:**
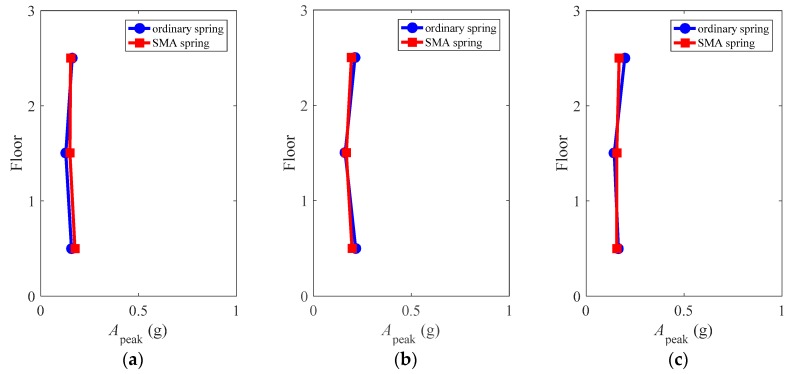
Peak floor acceleration of the superstructure: (**a**) LA01; (**b**) LA13; (**c**) LA17.

Figure 19 compares the height-wise peak floor accelerations of the superstructure, defined as:

(3) $$A_{peak}=\max\left(abs\left(A_{i}\left(t\right)\right)\right),\;i=1,2,...,6$$

where *A_i_* is the floor acceleration in the *i*th floor during the total time duration of *t*.

It can be seen that the isolated structures exhibited similar peak floor accelerations at each floor level, which implies increasing strength or adding damping at the isolator level generated similar effect to the floor acceleration demands. Besides, the magnitudes of the floor accelerations are very uniform from bottom to up, equaling to approximately 0.25 g, which is nearly one fourth of the up limitation defined by seismic provisions [35]. The assessment of floor accelerations indicates that both the ordinary spring and the SMA spring have excellent controllability for the floor acceleration demands of the frames.

### 4.9. Residual Interstory Drift Ratio

**Figure 20:**
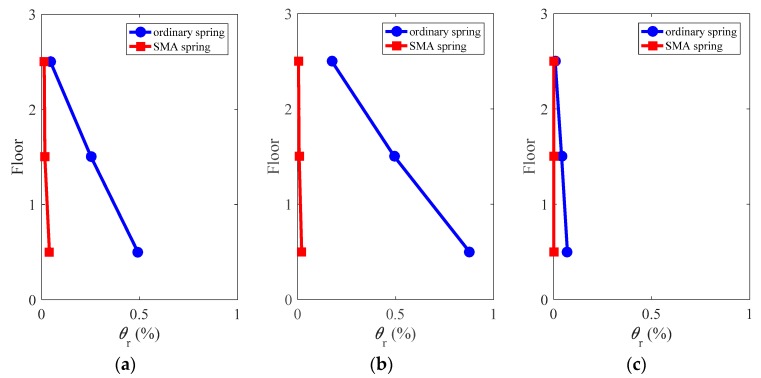
Residual interstory drift ratio of the superstructure: (**a**) LA01; (**b**) LA13; (**c**) LA17.

Figure 20 examines the profile of residual interstory drift ratios, defined as:

(4) $$\theta_{r}=abs\left(\theta_{i}\left(t=t_{end}\right)\right),\;i=1,2,...,6$$

where *θ_i_*(*t* = *t_end_*) is the interstory drift ratio in the *i*th story at the end of the total time duration of *t*.

The frame isolated with ordinary springs exhibited large residual deformation under the considered ground motions, particularly, under the ground motions of LA01 and LA13, the maximum residual interstory drift ratios are approximately 0.5% and 0.9%, which violates the threshold of devoting repairing effort after earthquakes [36]. When the frame is protected by SMA springs, however, the residual deformations markedly drop to almost zero along the height of the building under all considered ground motions. This shows that the installation of the SMA spring nearly eliminates the residual deformation of the frames, which renders the structure resilience after earthquakes.

## 5. Conclusions

This paper examined the seismic behavior of superelastic SMA spring in the isolation system of multi-story steel frame. According to the cyclic loading tests, the fabricated SMA spring exhibited desirable flag-shape hysteresis. The finite element model was calibrated by the test results and captured the hysteresis characteristics very well. Based on the finite element model built in OpenSees, seismic analyses were conducted by subjecting the isolated frames to selected earthquake ground motion records. Following conclusions can be obtained:

1. Superelastic SMA spring shows excellent self-centering capability; the equivalent damping ratio can be over 2%;
2. Good agreement can be found between the finite element model and test results;
3. The SMA spring has versatile cyclic properties, according to the parametric analyses on the geometrical dimensions;
4. Based on the comparisons with ordinary elastic spring, the advantages of using SMA spring in the isolation system mainly include the well control of the peak and residual deformations for the superstructure.

## Figures and Tables

**Table 1:** SMA material parameter used in finite element (FE) simulations.

E_A_	E_M_	σ_Ms_	σ_Mf_	σ_As_	σ_Af_	ε_t_
48 GPa	38 GPa	550 MPa	760 MPa	520 MPa	300 MPa	0.04

**Table 2:** Parameters of SMA springs.

Specimen No.	Parameters					
	*D* (mm)	*d* (mm)	*C*	*N*	Δ (mm)	*L* (mm)
S1	16	2	8	6	8	62
S2	16	3				
S3	16	4				
S4	18	2				
S5	20	2				

**Table 3:** Comparison of initial stiffness.

Stiffness (N/mm)	Specimens				
	S1	S2	S3	S4	S5
Simulation	2.01	10.69	33.85	1.41	0.99
Theory	1.97	9.97	31.52	1.38	1.01
Error	1.9%	6.7%	6.9%	2.1%	−1.9%

**Table 4:** Shape section data of the beams and columns.

Designation	Web Thickness (in)	Flange Width (in)	Flange Thickness (in)
W24 × 68	0.415	8.965	0.585
W21 × 44	0.350	6.500	0.450
W30 × 116	0.565	10.495	0.850
W33 × 118	0.550	11.480	0.740
W14 × 257	1.175	15.995	1.890
W14 × 311	1.410	16.230	2.260

**Table 5:** Material and geometrical information for the SMA and ordinary springs.

Spring Type	Length	Cross-Sectional Area	Elastic Modulus	“Yield” Stress	Post-Yield Stiffness Ratio	Hysteresis Width
SMA spring	0.4 m	344 mm^2^	50 Gpa	500 N/m^2^	0.26	0.5
Ordinary spring	0.4 m	344 mm^2^	-	-	-	-

**Table 6:** Considered ground motion records.

SAC Name	Record	Earthquake Magnitude	Distance (km)	Duration (s)	PGA (cm/s^2^)
LA01	Imperial Valley, 1940, El Centro	6.9	10	39.38	452.03
LA13	Northridge, 1994, Newhall	6.7	6.7	59.98	664.93
LA17	Northridge, 1994, Sylmar	6.7	6.4	59.98	558.43
